# Nonclassical Axis of the Renin-Angiotensin System and Neprilysin: Key Mediators That Underlie the Cardioprotective Effect of PPAR-Alpha Activation during Myocardial Ischemia in a Metabolic Syndrome Model

**DOI:** 10.1155/2020/8894525

**Published:** 2020-11-27

**Authors:** María Sánchez-Aguilar, Luz Ibarra-Lara, Leonardo del Valle-Mondragón, Elizabeth Soria-Castro, Juan Carlos Torres-Narváez, Elizabeth Carreón-Torres, Alicia Sánchez-Mendoza, María Esther Rubio-Ruíz

**Affiliations:** ^1^Department of Pharmacology, Instituto Nacional de Cardiología Ignacio Chávez, Juan Badiano 1, Sección XVI, Tlalpan, Mexico City 14080, Mexico; ^2^Department of Cardiovascular Biomedicine, Instituto Nacional de Cardiología Ignacio Chávez, Juan Badiano 1, Sección XVI, Tlalpan, Mexico City 14080, Mexico; ^3^Department of Molecular Biology, Instituto Nacional de Cardiología Ignacio Chávez, Juan Badiano 1, Sección XVI, Tlalpan, Mexico City 14080, Mexico; ^4^Department of Physiology, Instituto Nacional de Cardiología Ignacio Chávez, Juan Badiano 1, Sección XVI, Tlalpan, Mexico City 14080, Mexico

## Abstract

The activation of the renin-angiotensin system (RAS) participates in the development of metabolic syndrome (MetS) and in heart failure. PPAR-alpha activation by fenofibrate reverts some of the effects caused by these pathologies. Recently, nonclassical RAS components have been implicated in the pathogenesis of hypertension and myocardial dysfunction; however, their cardiac functions are still controversial. We evaluated if the nonclassical RAS signaling pathways, directed by angiotensin III and angiotensin-(1-7), are involved in the cardioprotective effect of fenofibrate during ischemia in MetS rats. Control (CT) and MetS rats were divided into the following groups: (a) sham, (b) vehicle-treated myocardial infarction (MI-V), and (c) fenofibrate-treated myocardial infarction (MI-F). Angiotensin III and angiotensin IV levels and insulin increased the aminopeptidase (IRAP) expression and decreased the angiotensin-converting enzyme 2 (ACE2) expression in the hearts from MetS rats. Ischemia activated the angiotensin-converting enzyme (ACE)/angiotensin II/angiotensin receptor 1 (AT1R) and angiotensin III/angiotensin IV/angiotensin receptor 4 (AT4R)-IRAP axes. Fenofibrate treatment prevented the damage due to ischemia in MetS rats by favoring the angiotensin-(1-7)/angiotensin receptor 2 (AT2R) axis and inhibiting the angiotensin III/angiotensin IV/AT4R-IRAP signaling pathway. Additionally, fenofibrate downregulated neprilysin expression and increased bradykinin production. These effects of PPAR-alpha activation were accompanied by a reduction in the size of the myocardial infarct and in the activity of serum creatine kinase. Thus, the regulation of the nonclassical axis of RAS forms part of a novel protective effect of fenofibrate in myocardial ischemia.

## 1. Introduction

The renin-angiotensin system (RAS) is a complex hormone system that plays a critical role in cardiovascular physiology. Indeed, RAS has a central role in the development of metabolic syndrome (MetS), insulin resistance, and heart failure [1].

The RAS is a system composed of different angiotensin peptides with different biological actions mediated by distinct receptor subtypes. The classical RAS comprises the renin angiotensin-converting enzyme (ACE)/angiotensin II (Ang II)/angiotensin receptor 1 (AT1R) axis and promotes vasoconstriction and increases oxidative stress, fibrosis, cellular growth, and inflammation [1]. In contrast, the nonclassical RAS, composed mainly by the angiotensin-converting enzyme 2 (ACE2)/angiotensin-(1-7) (Ang-(1-7))/Mas receptor (MasR)/angiotensin receptor 2 (AT2R) pathway, improves the cardiac function of hearts subjected to myocardial infarction (MI) and has a beneficial role in insulin resistance, hypertriglyceridemia, fatty liver disease, and obesity [1–4].

Currently, additional metabolites of RAS and some of their biological functions have been described. Ang III, formed from Ang II by aminopeptidase A (APA), is cleaved by aminopeptidase N (APN) forming Ang IV. Ang IV binds to the angiotensin type 4 receptor (AT4), identified as an insulin-regulated aminopeptidase (IRAP), and it plays a potential role in the regulation of glucose homeostasis and inflammatory processes and in the metabolism of various hormones including vasopressin, oxytocin, and somatostatin [5, 6]. Ang III and APA are potential therapeutic targets for the treatment of hypertension; however, the roles of Ang III and Ang IV in cardiac function remain controversial [7–10].

On the other hand, neprilysin (NEP) is a ubiquitous endopeptidase of RAS, essential for the metabolism of the biologically active natriuretic peptides and several other vasoactive compounds, including adrenomedullin, endothelin 1, and bradykinin [11]. Moreover, plasmatic NEP levels have been positively associated with heart failure, obesity, and MetS [12, 13]. This enzyme is a target of multiple clinical trials given its importance in cardiovascular diseases, and a new class of drugs called “ARNI” (angiotensin receptor blocker-neprilysin inhibitor) is currently being used as therapy for hypertension and heart failure [14, 15].

Fenofibrate acts as an agonist of the peroxisome proliferator-activated receptor alpha (PPAR-*α*) that regulates the expression of target genes, including some components of RAS. Fenofibrate has pleiotropic effects besides lowering lipids, such as improving vascular endothelial function and reducing oxidative stress, inflammation, and fibrosis during cardiac ischemia [1, 16, 17]. However, the exact mechanism underlying the beneficial effect of fenofibrate on cardiovascular diseases remains uncertain.

Our previous work demonstrated that fenofibrate treatment decreased ischemic damages by favoring an antioxidant environment as a consequence of reducing the Ang II/AT1R signaling pathway and reestablishing the cardiac insulin signaling pathway. Although numerous experimental studies have focused on an alternative RAS cascade, to our knowledge, there are no reports on the effect of fenofibrate on these novel important components of RAS in myocardial ischemia. Therefore, the aim of the current study was to evaluate if the nonclassical RAS signaling pathways, directed by Ang III, Ang-(1-7), and NEP expression, are involved in the cardioprotective effect of fenofibrate during ischemia in MetS rats.

## 2. Methods

### 2.1. Animals and Surgical Procedures

All the experiments were conducted in accordance with our Institutional Ethical Guidelines (Ministry of Agriculture, SAGARPA, NOM-062-ZOO-1999, Mexico). Male 25-day-old Wistar rats weighing 45 ± 9 g were randomly separated into two groups of 10-12 animals: group 1, control rats that were given tap water for drinking; group 2, MetS rats that received 30% sugar in drinking water during 24 weeks. The animals were maintained under standard conditions of light and temperature with water and food *ad libitum* (LabDiet 5001; Richmond, IN, USA). Systolic arterial blood pressure was determined in conscious animals by a plethysmographic method described previously [1].

Animals from each experimental group were divided to receive one of the subchronic (two weeks) oral gavage treatments: (a) vehicle (NaCl 0.9%) or (b) fenofibrate (100 mg/kg/day). This dose was selected based on previous publications and from a dose-response curve to fenofibrate [1, 16, 17]. At the end of the treatment, the animals were anesthetized (ketamine hydrochloride 80 mg/kg and xylazine hydrochloride 10 mg/kg, I.M.) and they were assigned to either sham-operated (Sh) or myocardial infarction (MI) for 60 min. As previously reported, MI was achieved occluding the left anterior descending coronary artery with 7-0 PROLENE® polypropylene suture (Ethicon, São José dos Campos, Brazil) [1]. Then, the rats were sacrificed, the heart was cut out, and the ischemic area was separated to perform the analysis. Additionally, the abdominal white adipose tissue was removed and weighed.

### 2.2. Measurement of Serum Biochemical Parameters

The fasting measurements of glucose, HDL-C, non-HDL-C, and triglycerides were performed with commercial enzymatic kits. Serum insulin levels were measured using a rat-specific insulin radioimmunoassay (Linco Research, Inc., Missouri, USA). Insulin resistance was estimated from the homeostasis model (HOMA-IR) [18].

Creatine kinase (CK) activity was spectrophotometrically determined at 340 nm (UV-test, Roche Cobas C-501, Roche Diagnostics, Indianapolis, IN, USA) after the MI procedure. The determination of CK was carried out using the reverse reaction and activation by N-acetylcysteine (NAC) at 37°C. Equimolar quantities of NADPH and ATP are formed at the same rate. The photometrically measured rate of formation of NADPH is directly proportional to the CK activity [19].

### 2.3. Electrophoretic Determinations

Ang II, Ang III, Ang IV, and Ang-(1-7) concentrations were evaluated in myocardial ischemic areas from the different experimental groups by capillary zone electrophoresis (CZE, P/ACE MDQ Capillary Electrophoresis System, Beckman Coulter, Inc., Fullerton, CA, USA) according to the methods previously described [1, 18]. Bradykinin was evaluated by capillary electrophoresis with a laser-induced fluorescence detector, as previously reported [20].

### 2.4. Western Blotting Analysis

The frozen myocardial ischemic area was homogenized with a polytron (model PT-MR2100; Kinematica AG, Lucerne, Switzerland) (25% *w*/*v*) in a lysis buffer pH = 7.4 (Tris-HCl 250 mM, NaF 0.2 M, Na_3_VO_4_ 10 mM, and NP40 1%) and a protease inhibitor cocktail (cOmplete® tablets, Roche Applied Science, Mannheim, Germany) at 4°C. A total of 100 *μ*g protein was separated on a SDS-PAGE (12% bis-acrylamide-Laemmli gel) and transferred to a polyvinylidene difluoride (PVDF) membrane. Blots were blocked for 3 h at room temperature using Tris-buffered saline with 0.05% Tween (TBS-T) and 5% nonfat dehydrated milk. Afterwards, membranes were incubated overnight with primary antibodies at 4°C. ACE, ACE2, AT1R, AT2R, MasR, APA, APN, and NEP primary antibodies were acquired from Santa Cruz Biotechnology (Santa Cruz, CA, USA); IRAP antibody was from Cell Signaling Technology (Danvers, MA, USA). Secondary horseradish peroxidase-labeled antibodies were from Jackson ImmunoResearch (Suffolk, UK). All blots were incubated with *β*-actin antibody as a load control. After incubation, the blots were visualized using the Immobilon chemiluminescent system (Immobilon Western, Millipore, MA, USA) [1]. Images from films were digitally obtained using a GS-800 densitometer with the Quantity One software (Bio-Rad Laboratories, Inc.) and are reported as arbitrary units (AU).

### 2.5. Determination of the Infarct Size

After 60 min of ischemia, the rats were euthanized, and the hearts were rapidly excised. Later, the hearts were perfused with 1.5 mL of 0.05% Evans blue dye on the Langendorff system; this procedure was performed to outline the ischemic myocardium (and area at risk). The hearts were frozen at -20°C for 1 hour, and then 2 mm thick cross-sections were obtained. The slices were covered completely with 2,3,5-triphenyl tetrazolium hydrochloride (TTC) at 1% in phosphate-buffered saline (PBS) (1 M, pH 7.2) and incubated for 20 min at 37°C to distinguish the viable myocardium from the necrotic tissue. Later, the slices were incubated in 10% formalin for one hour and maintained in PBS until the image was obtained. The slices were photographed to show the ischemic area with a Multiphot Canon camera EOS 6D (Tokyo, Japan) [17].

### 2.6. Statistical Analysis

Results are expressed as mean ± standard error of the mean (SEM). Experimental data were examined using the one-way ANOVA followed by the Newman-Keuls *post hoc* test. Differences were considered statistically significant when *p* < 0.05. All analyses were performed using the statistical package GraphPad Prism version 5.03 (GraphPad Software, La Jolla, CA).

## 3. Results

The characterization of the MetS model was done by analyzing the animal's body weight, blood pressure, and intra-abdominal fat and by the serum biochemical analysis. As shown in Table 1, MetS animals developed central obesity, hypertension, dyslipidemia (high levels of triglycerides and non-HDL-C and low levels of HDL-C), hyperinsulinemia, and insulin resistance (HOMA-IR).

As expected, the treatment with fenofibrate significantly reduced the concentration of triglycerides and non-HDL-C levels and restored the insulin resistance index (HOMA-IR) in the MetS group. In the CT group, fenofibrate administration significantly reduced the concentration of non-HDL-C and did not affect the other parameters.

Serum CK activity was determined in all groups. No significant difference was found between the CT and MetS sham-operated groups (533.30 ± 48.14 vs. 626.50 ± 101.40). After MI, CK activity was significantly higher in MetS than in CT rats treated with vehicle (Table 1). Fenofibrate treatment significantly reduced CK activity in serum from the CT and MetS animals (23% and 14%, respectively).

**Figure 1:**
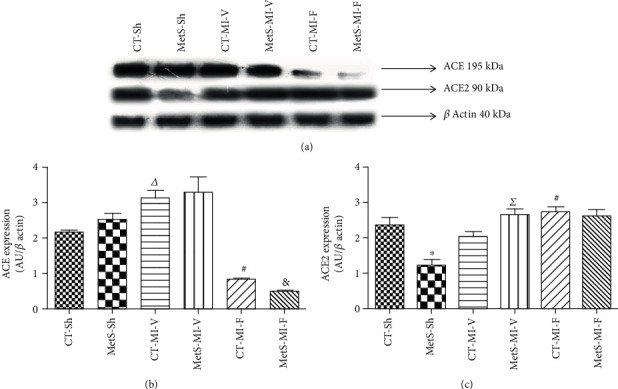
Effect of fenofibrate on the expression of renin-angiotensin-converting enzyme (ACE) and ACE2. The expression was evaluated in the left ventricles from the control (CT) and metabolic syndrome (MetS) rats subjected to sham (Sh) or myocardial infarction (MI) and treated for two weeks with either vehicle (V) or fenofibrate (F). (a) Representative western blot analysis. (b) ACE protein expression. (c) ACE2 protein expression. Data represent mean ± SEM (*n* = 5 per group). ^∗^*p* < 0.05 vs. CT-Sh; *^Δ^p* < 0.05 vs. CT-Sh; *^Σ^p* < 0.05 vs. MetS-Sh; ^#^*p* < 0.05 vs. CT-MI-V; ^&^*p* < 0.05 vs. MetS-MI-V. Analysis of variance-Newman-Keuls.

Figure 1 shows the expression of ACE and ACE2 in the homogenate from the left ventricles from each experimental group. ACE expression was slightly higher in the MetS group than in CT. Ischemia promoted an increase in ACE expression in the CT group, while the expression remained essentially unchanged in hearts from MetS rats. Fenofibrate treatment significantly diminished ACE expression in the same proportion in both experimental groups (Figures 1(a) and 1(b)).

Due to the relevance of ACE2 for the production of Ang-(1-7), we studied whether fenofibrate exerts an effect on this enzyme. ACE2 expression was significantly decreased in the MetS group compared to the CT group (Figure 1(c)). When the hearts were subjected to ischemia, ACE2 increased in the MetS group and no change was observed in CT rats. The administration of fenofibrate significantly increased ACE2 expression in the CT group and had no effect on MetS animals.

**Figure 2:**
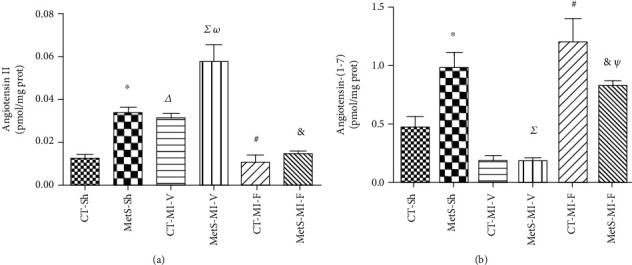
Effect of fenofibrate on angiotensin II and angiotensin-(1-7) levels. (a) Angiotensin II and (b) angiotensin-(1-7) concentrations were determined in the homogenate from myocardial ischemic areas from the sham (Sh), myocardial infarction (MI) vehicle-treated, and MI fenofibrate-treated experimental groups. The values show the mean ± SEM (*n* = 5 per group). ^∗^*p* < 0.05 vs. CT-Sh; *^Δ^p* < 0.05 vs. CT-Sh; *^Σ^p* < 0.05 vs. MetS-Sh; ^#^*p* < 0.05 vs. CT-MI-V; *^ω^p* < 0.05 vs. CT-MI-V; ^&^*p* < 0.05 vs. MetS-MI-V; *^Ψ^p* < 0.05 vs. CT-MI-F. Analysis of variance-Newman-Keuls.

According to the pathophysiology, the left ventricular Ang II levels increased in MetS rats in comparison to CT rats (Figure 2(a)). When the hearts were under ischemic conditions, Ang II levels increased in CT and there was a further increase in MetS rats; however, fenofibrate administration significantly diminished Ang II concentrations. Importantly, in the MetS group, the Ang-(1-7) levels were significantly higher in basal conditions, but under ischemic conditions, Ang-(1-7) levels decreased (Figure 2(b)). Fenofibrate treatment significantly increased the concentration of this peptide in both groups (Figure 2(b)).

**Figure 3:**
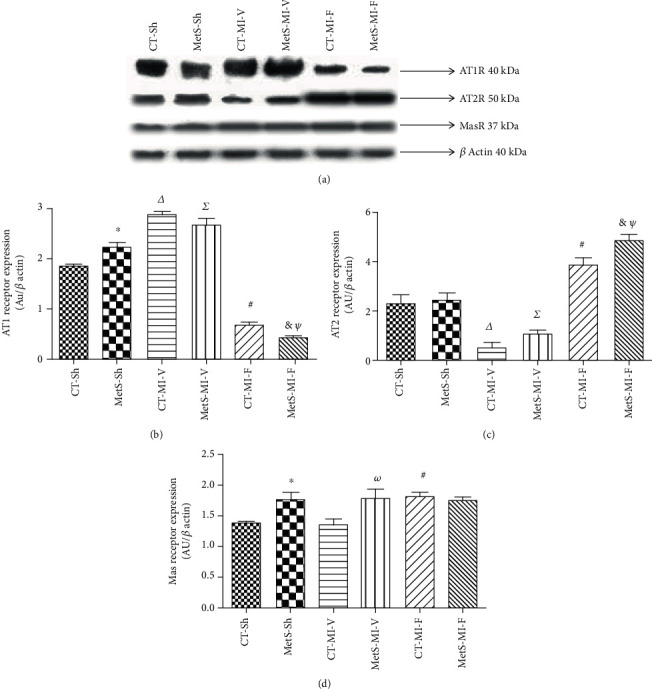
Expression of cardiac angiotensin receptors. The proteins were evaluated in the left ventricles from the control (CT) and metabolic syndrome (MetS) rats subjected to sham (Sh) or myocardial infarction (MI) and treated for two weeks with either vehicle (V) or fenofibrate (F). (a) Representative immunoblot. (b) Angiotensin II-type 1 receptor (AT1R) protein expression. (c) Angiotensin II-type 2 receptor (AT2R) protein expression. (d) MasR protein expression. The values show the mean ± SEM (*n* = 5 per group). ^∗^*p* < 0.05 vs. CT-Sh; *^Δ^p* < 0.05 vs. CT-Sh; *^Σ^p* < 0.05 vs. MetS-Sh; ^#^*p* < 0.05 vs. CT-MI-V; *^ω^p* < 0.05 vs. CT-MI-V; ^&^*p* < 0.05 vs. MetS-MI-V; *^Ψ^p* < 0.05 vs. CT-MI-F. Analysis of variance-Newman-Keuls.

Western blot analyses revealed differences in the expression of AT1R, AT2R, and MasR in hearts from the CT and MetS groups (Figures 3(a)–3(d)). As expected, AT1R expression was higher in MetS rats compared to CT rats. Ischemia promoted an increase in AT1 expression in both the CT and MetS groups. Fenofibrate treatment significantly diminished the AT1R expression in both groups, although this effect was more evident in the MetS rats (Figures 3(a) and 3(b)). Results show that AT2R expression did not differ between the two sham-operated groups. When the hearts were subjected to ischemia, a significant decrease of AT2R expression in the CT and MetS groups was observed. The administration of fenofibrate significantly increased the expression of this receptor in both groups (Figures 3(a) and 3(c)). MasR expression was significantly higher in MetS-Sh compared to CT-Sh animals; the expression of this receptor under ischemic conditions was comparable to values from sham experimental groups. The treatment with fenofibrate promoted an increase in MasR expression in CT rats, while its expression remained unchanged in hearts from MetS (Figures 3(a) and 3(d)).

**Figure 4:**
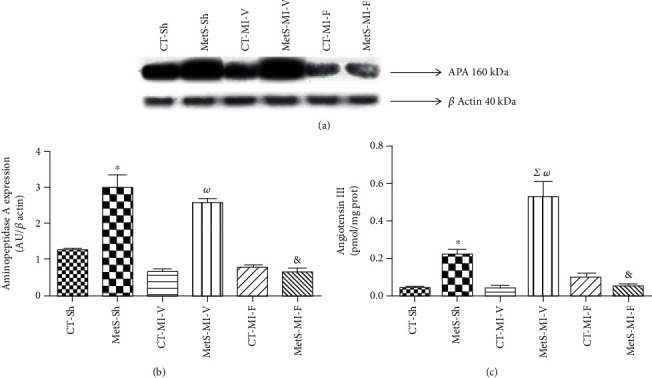
Effect of fenofibrate on the expression of aminopeptidase A (APA) and angiotensin III (Ang III) concentrations in the control (CT) and metabolic syndrome (MetS) rats under ischemic conditions (MI). (a) Representative western blot analysis. (b) APA protein expression. (c) Ang III concentration. Arbitrary units (AU). The values show the mean ± SEM (*n* = 5 per group). ^∗^*p* < 0.05 vs. CT-Sh; *^Σ^p* < 0.05 vs. MetS-Sh; *^ω^p* < 0.05 vs. CT-MI-V; ^&^*p* < 0.05 vs. MetS-MI-V. Analysis of variance-Newman-Keuls.

Since Ang III and Ang IV have been identified as new biologically active peptides of RAS, we studied the effect of fenofibrate on the production of these metabolites. Figures 4(a)–4(c) show that the expression of APA (the major enzyme metabolizing Ang II to Ang III) and Ang III concentration were significantly increased in the left ventricle of MetS-Sh animals compared to CT-Sh. This effect was also observed under ischemic conditions. Fenofibrate was able to prevent the increase in APA and Ang III levels in MetS animals.

**Figure 5:**
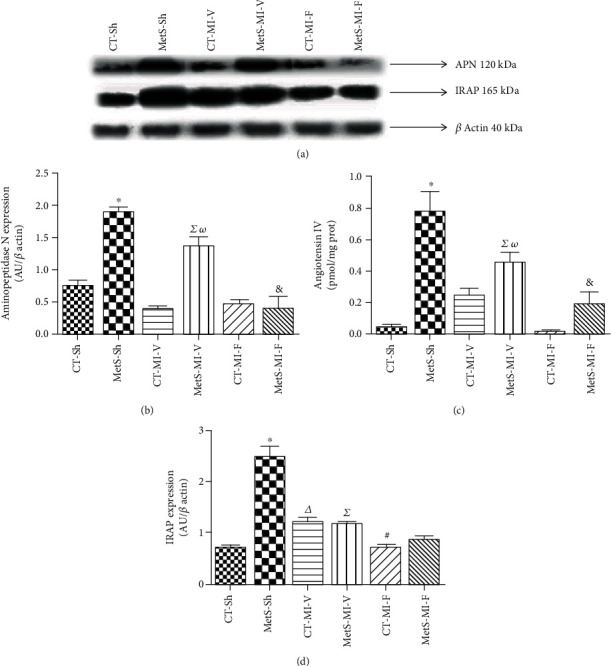
Effect of fenofibrate administration on myocardial aminopeptidase N (APN) expression in myocardial tissue from the control (CT) and metabolic syndrome (MetS) animals. (a) Representative image. (b) Angiotensin IV concentration. (c) Insulin-regulated aminopeptidase (IRAP) protein expression. The values show the mean ± SEM (*n* = 5 per group). ^∗^*p* < 0.05 vs. CT-Sh; *^Δ^p* < 0.05 vs. CT-Sh; *^Σ^p* < 0.05 vs. MetS-Sh; ^#^*p* < 0.05 vs. CT-MI-V; *^ω^p* < 0.05 vs. CT-MI-V; ^&^*p* < 0.05 vs. MetS-MI-V. Analysis of variance-Newman-Keuls.

We also investigated if fenofibrate induced variations in Ang III concentrations which might be associated with the Ang IV-IRAP pathway. Our results showed that hearts from MetS-Sh had significantly higher levels of APN, Ang IV, and IRAP when compared to the corresponding CT group (Figures 5(a)–5(d)). Ischemia was accompanied by a decrease in the components analyzed in hearts from MetS rats; however, the levels of APN and Ang IV did not change significantly in the CT group. Fenofibrate treatment was able to prevent the activation of the Ang IV-IRAP axis in the left ventricles from MetS rats (Figures 5(a)–5(d)).

**Figure 6:**
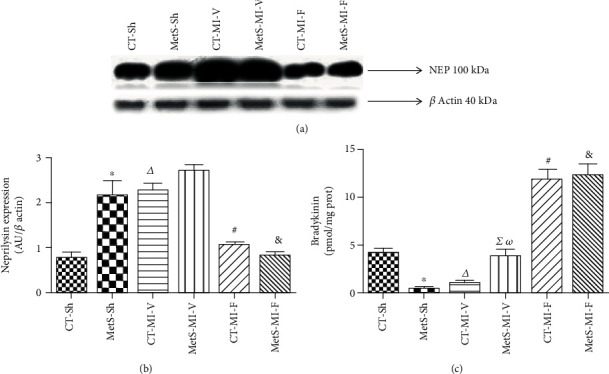
Effect of fenofibrate on the expression of neprilysin (NEP) and bradykinin concentration in control and under ischemic conditions. (a) Representative western blot analysis. (b) NEP protein expression. (c) Bradykinin concentration. The values show the mean ± SEM (*n* = 5 per group). ^∗^*p* < 0.05 vs. CT-Sh; *^Δ^p* < 0.05 vs. CT-Sh; *^Σ^p* < 0.05 vs. MetS-Sh; ^#^*p* < 0.05 vs. CT-MI-V; *^ω^p* < 0.05 vs. CT-MI-V; ^&^*p* < 0.05 vs. MetS-MI-V. Analysis of variance-Newman-Keuls.

Due to the beneficial effects of the inhibition of NEP and the cardioprotective effects of bradykinin, we evaluated the effect of fenofibrate on NEP expression and bradykinin production. Figures 6(a) and 6(b) show that under basal conditions, MetS hearts expressed more NEP when compared to CT hearts.

In CT-MI vehicle-treated animals, the expression of this enzyme increased under ischemic conditions; nevertheless, in the MetS-MI vehicle-treated group, NEP levels were not modified. The concentrations of bradykinin showed the opposite effect (Figure 6(c)). The administration of fenofibrate was associated with a significant decrease in NEP expression and a consequent increase in bradykinin production in both groups.

**Figure 7:**
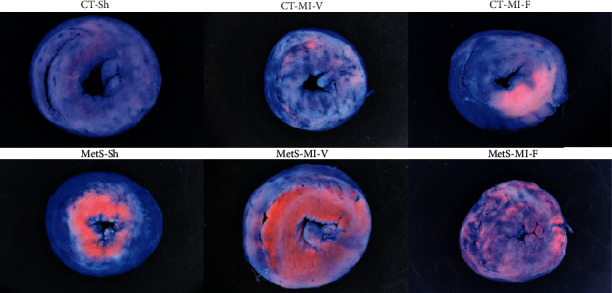
Fenofibrate treatment decreased the area at risk in myocardial infarcted rats. Representative images of TTC staining. Blue areas indicate viable tissue; the red-colored region represents tissue at risk, and the white regions indicate the infarcted myocardium. CT-Sh and MetS-Sh: control and metabolic syndrome sham-operated groups; CT-MI-V: control myocardial infarction vehicle-treated; CT-MI-F: control myocardial infarction fenofibrate-treated; MetS-MI-V: metabolic syndrome myocardial infarction vehicle-treated; MetS-MI-F: metabolic syndrome myocardial infarction fenofibrate-treated.

The infarct size was evaluated using Evans blue dye plus TTC at 1%. Blue-dyed myocardial tissue represents viable tissue. Left ventricles obtained from the CT-Sh group exhibit an extensive blue area. Tissue from MetS-Sh shows a blue periphery that signals the border of the central zone colored in red. The red area represents the area at risk (Figure 7). In MI groups treated with vehicle, the TCC-stained heart slice confirms infarcted areas (white spots within the viable area); nevertheless, the area at risk (red-colored region) was greater in hearts from MetS rats. The treatment with fenofibrate attenuated the tissue injury in the ischemic area of the MetS and CT rats. The improvement was larger in the CT group where the area at risk and ischemic regions decreased (limited at the central area). However, these areas were widely distributed in the tissue in hearts from MetS rats (Figure 7).

## 4. Discussion

This work shows that fenofibrate treatment generates cardioprotection in an experimental model of MetS subjected to ischemia, by regulating the nonclassical pathways of RAS. Our study demonstrated that the pharmacological treatment was associated with the activation of Ang-(1-7)/AT2R and inhibition of Ang III/Ang IV/IRAP pathways. Moreover, another novel finding of this study was that the treatment with fenofibrate may decrease the expression of NEP with the consequent increase of bradykinin production.

Extensive research has revealed that the activation of specific molecules of RAS participates in the development of MetS and heart failure. Fenofibrate therapy reverts some of the effects caused by these pathologies by regulating several processes mediated by Ang II/AT1, such as energy metabolism, oxidative stress, inflammation, and cell differentiation [1]. On the other hand, an accumulating amount of data signals the importance of an alternative pathway of RAS, such as Ang-(1-7) and its cardioprotective properties. Our data are in line with previous findings; however, there are no reports on the effect of fenofibrate on the important counterparts of the RAS pathways analyzed in this paper as far as we know.

Data in Table 1 show that the fenofibrate treatment reversed the signs of MetS such as dyslipidemia and insulin resistance, and it did not affect the other parameters. These data are in accordance with those previously published [1].

Serum CK activity is well known as a cardiac risk biomarker in human and animal models; however, this parameter is unspecific [21–23]. The results in Table 1 show that CK activity was higher in serum from MetS-MI rats than the CT-MI group treated with vehicle. In addition, we demonstrated that fenofibrate treatment significantly reduced the increase of CK in the serum from both the CT and MetS rats. Nevertheless, this effect was more evident in the CT group. Therefore, our results show that CK activity is a good biomarker of myocardial damage in our experimental model.

In this study, the classical and nonclassical RAS axes were studied. The ACE/Ang II/AT1R axis has been the main RAS pathway studied, and its roles in cardiac damage and therapeutic implications have been extensively reviewed [1]. As expected, the ACE expression and the Ang II concentration were higher (slightly and significantly, respectively) in hearts from MetS animals compared to the CT-Sh group. Ischemic conditions were associated with an increase in the expression of the enzyme and its product (Figures 1(a) and 2(a)). Fenofibrate treatment prevented the increase of ACE expression in both the CT and MetS groups. Consistent with these findings, the Ang II concentration decreased in both experimental groups (Figures 1(b) and 2(a)). As far as we know, there are no reports on the determination of Ang II levels in patients undergoing fenofibrate therapy. There are conflicting data regarding the effect of PPAR agonists on blood pressure in humans, due to heterogeneity of populations, the mechanism underlying the high blood pressure, and differences in concurrent treatments or dietary salt intakes [24]. However, Walker et al. [25] demonstrated a beneficial effect of fenofibrate on vascular endothelial function in humans. Fenofibrate reduces oxidative stress and increases eNOS expression. These effects might be related to the reduction of Ang II concentrations and agree with our previously reported results [1].

On the other hand, ACE2 efficiently hydrolyses Ang II to form Ang-(1-7), a peptide that exerts opposite actions to those of Ang II. Our results show that under basal conditions, the expression of ACE2 was not accompanied by similar changes in Ang-(1-7) concentration. Surprisingly, the ischemic insult promoted an increase in ACE2 expression while the levels of Ang-(1-7) decreased in the MetS group (Figures 1(c) and 2(b)). The increase in ACE2 expression under ischemia conditions suggests a protective role of this enzyme and is in agreement with the data published by other authors in human and animal models [26, 27]. However, the activity of ACE2 should be evaluated to explain the discrepancy between ACE2 expression and Ang-(1-7) concentration. This constitutes a limitation of the present study. The levels of Ang-(1-7) were significantly higher in the ischemic hearts in the presence of fenofibrate in both the CT and MetS animals (Figure 2(b)). Ang-(1-7) decreases hypertension, cardiac hypertrophy, oxidative stress, and insulin resistance [28, 29]. Furthermore, in a previous report, we demonstrated that clofibrate treatment increased Ang-(1-7) concentration in the ischemic myocardium [20].

Figure 3 shows that there is an overexpression of AT1R and MasR in hearts from MetS rats under basal conditions. Under ischemic conditions, AT1R levels increased and AT2R levels decreased in both experimental groups; fenofibrate therapy reversed this effect. The expression of Mas increased only in the CT-MI-F group (Figures 3(a)–3(d)). It has been reported that Ang-(1-7) interacts with both MasR and AT2R [30–32]. Therefore, it is reasonable to suggest that the cardioprotective actions could be mediated through these receptors.

Altogether, our results show that ischemia promotes the activation of the ACE/Ang II/AT1R pathway and that the activation of the Ang-(1-7)/MasR-AT2 signaling pathway is involved in the cardioprotective role of fenofibrate.

Subsequently, we studied the effect of fenofibrate therapy on the nonclassical components of the RAS pathway. Two smaller angiotensin peptides, Ang III and Ang IV, have been reported to have harmful effects via AT1R activation [33, 34]. Ang III and Ang IV levels were increased in the left ventricles in MetS-Sh rats (Figures 4(c) and 5(c)). After the ischemic insult, Ang III concentrations were significantly increased, while Ang IV levels diminished in MetS vehicle-treated hearts. We suggest that this may be a compensatory effect to the ischemic damage. These results were consistent with the expression of APA and APN, respectively (Figures 4(b) and 5(b)). The administration of fenofibrate prevents the rise in the small peptides in MetS rats, while the values remained unchanged in CT animals. Clearly, the decrease in the concentration of the small peptides was directly related to the decrease of its precursor, Ang II; however, our results show that the administration of fenofibrate had a direct association with the expression of APA and APN. Moreover, the receptor to Ang IV has been identified in several tissues as an IRAP. The presence of IRAP is important for the translocation of the insulin-stimulated GLUT4 and in the metabolism of oxytocin [33, 35, 36]. MetS and ischemic conditions were associated with IRAP overexpression, and the therapy with fenofibrate decreased the levels of this protein (Figure 5(d)).

Ang IV is the endogenous inhibitor of the catalytic binding site of IRAP preventing the metabolism of various IRAP substrates, including oxytocin. Oxytocin exerts cardioprotection, either directly or via stimulation of mediators such as the natriuretic peptides and nitric oxide [37]. Furthermore, this hormone enhances glucose uptake via the translocation of GLUT4 to the cell surface [36, 38]. Therefore, we speculate that the Ang IV/AT4R/IRAP axis is also involved in cardiac insulin resistance under ischemic conditions, regulating oxytocin concentrations. Fenofibrate treatment might improve glucose uptake by increasing oxytocin levels as a consequence of the downregulation of the IRAP expression. These suggestions are in agreement with our previous study, in which we showed that ischemia impairs myocardial insulin action and that fibrates reestablish the cardiac insulin signaling pathway [1]. There are few studies reporting that the IRAP deficiency/inhibition protects against cardiac damage; therefore, further studies demonstrating the pathophysiological role of the Ang IV/AT4R/IRAP axis are needed.

We studied whether the administration of fenofibrate had an effect on the expression levels of NEP and bradykinin, due to the interaction between the RAS and the NEP system and the role of peptides, such as bradykinin, in heart homeostasis. We observed that MetS and ischemic conditions were accompanied by a NEP overexpression with the consequent reduction in the concentration of bradykinin (Figures 6(b) and 6(c)). Fenofibrate treatment favored the production of bradykinin by suppressing NEP expression in hearts from both the CT and MetS rats. Our findings suggest the involvement of NEP in the cardioprotective effect of fenofibrate, and this effect could be mediated by genomic actions through the previously reported PPAR activation [39]. Thus, the increase in bradykinin levels might mediate several cardiac functions, as has been previously reported by other authors [40, 41]. Further studies are needed to prove the effect of the pretreatment with fenofibrate on the production of other hormones metabolized by NEP during heart failure, such as natriuretic peptides.

Finally, in order to evaluate the efficacy of our treatment, we analyzed the ischemic size by the TTC staining analysis. Evans blue dye has been used extensively to stain hearts, and it accurately reflects the extent of viable areas and irreversible myocardial ischemic damage.  Figure 7 shows that hearts from MetS-Sh rats present a larger area at risk when compared to the CT group. Our results are in line with experimental and clinical trial evidence which suggests that MetS is associated with myocardial infarction [42]. After MI insult, the MetS heart had a larger area at risk and infarcted area when compared to the heart from CT rats. Fenofibrate treatment was able to attenuate MI-induced damage evidenced by the reduction of the area at risk and ischemic area (red- and white-colored regions). This effect is more evident in the CT group. The results of our work are in agreement with those of Mo et al. [22], who showed that fenofibrate reduced the myocardial infarct size.

Cardiac myocytes in the ischemic zone die by apoptosis and necrosis during a myocardial infarction; nevertheless, the magnitudes of each form of cell death remain unclear. Krijnen et al. [43] described that upon permanent occlusion of a coronary vessel in rats, apoptosis occurred in the ischemic region, in the area immediately bordering the ischemic region, and in remote regions from ischemia. Therefore, apoptosis is the major determinant of infarct size. Necrosis occurred less often and was seen only in the ischemic region.

Nevertheless, we did not perform an analysis of cell necrosis and apoptosis in the present study. In a previous report of our group, we found that PPAR-*α* stimulation with clofibrate augmented the expression of antiapoptotic proteins Bcl-2 and 14-3-3*ε* and decreased the expression of proapoptotic proteins Bax and the phosphorylation of Bad [44]. Moreover, oxidative stress coexists with apoptosis after myocardial infarction [43]. Our model also presents oxidative stress [1], and therefore, we cannot exclude an effect similar to the one previously reported by our group.

## 5. Conclusion

Overall, our results show that PPAR-alpha activation by fenofibrate prevents damage due to ischemia in MetS rats by changing the angiotensin metabolites and their receptor profiles. It favors the Ang-(1-7)/AT2 axis and inhibits the Ang III/Ang IV/IRAP signaling pathway. In addition, PPAR-alpha signaling downregulates the expression of NEP and increases the production of bradykinin. Thus, the regulation of the nonclassical axis of RAS is a novel protective effect to myocardial ischemia of fenofibrate.

## Figures and Tables

**Table 1:** The effects of fenofibrate administration on body characteristics and biochemical parameters from the control (CT) and MetS rats.

	CT-V	CT-F	MetS-V	MetS-F
Body weight (g)	503.0 ± 18.3	490.5 ± 19.8	518.7 ± 14.6	502.5 ± 13.5
Blood pressure (mmHg)	98.6 ± 5.8	101.5 ± 1.3	148.1 ± 6.5^a^	141.5 ± 11.3^a^
Intra-abdominal fat (g)	6.4 ± 0.7	5.2 ± 0.5	13.4 ± 0.8^a^	12.9 ± 0.9
Triglycerides (mg/dL)	60.8 ± 11.3	48.8 ± 5.3	135.6 ± 10.9^a^	53.7 ± 10.2^b^
HDL-C (mg/dL)	42.5 ± 4.8	41.7 ± 1.3	21.8 ± 3.9^a^	23.5 ± 5.8
Non-HDL-C (mg/dL)	19.9 ± 2.1	11.7 ± 1.8^b^	34.8 ± 2.8^a^	12.8 ± 2.6^b^
Total cholesterol (mg/dL)	58.6 ± 4.3	52.5 ± 3.8	61.7 ± 1.2	48.8 ± 6.4
Glucose (mg/dL)	101.3 ± 6.7	98.5 ± 3.2	110.6 ± 10.3	100.4 ± 5.8
Insulin (ng/mL)	0.16 ± 0.05	0.11 ± 0.03	0.39 ± 0.06^a^	0.14 ± 0.07^b^
HOMA-IR	1.1 ± 0.2	0.9 ± 0.1	4.1 ± 0.9^a^	1.6 ± 0.7^b^
CK activity (U/L)	1556.0 ± 77.77	1190.33 ± 73.12^c^	1964.0 ± 52.03^a^	1688.6 ± 54.18^a,c^

Values are mean ± SEM. The serum biochemical determinations were performed after the myocardial insult. CT-V: control vehicle-treated; CT-F: control fenofibrate-treated; MetS-V: metabolic syndrome vehicle-treated; MetS-F: metabolic syndrome fenofibrate-treated; HOMA-IR: homeostatic model assessment of insulin resistance; HDL-C: high-density lipoprotein cholesterol; CK: creatine kinase. *n* = 6. ^a^*p* < 0.01 MetS vs. CT same treatment; ^b^*p* < 0.05 against vehicle corresponding group; ^c^*p* < 0.01 against vehicle corresponding group.

## Data Availability

The data in our study are available from the corresponding author upon reasonable request.
